# Spectroscopic contribution to glyphosate toxicity profile and the remedial effects of *Momordica charantia*

**DOI:** 10.1038/s41598-022-24692-7

**Published:** 2022-11-21

**Authors:** Emine Yalçin, Kültiğin Çavuşoğlu

**Affiliations:** Department of Biology, Faculty of Arts and Sciences, Giresun University, 28200 Giresun, Turkey

**Keywords:** Cell biology, Genetics, Plant sciences

## Abstract

In this study, the glyphosate toxicity and the toxicity-reducing role of bitter melon extract (Bmex) (*Momordica charantia* L.) were investigated in *Allium cepa* L. test material. The toxicity of glyphosate and protective role of Bmex were investigated with the help of physiological (germination, root elongation and weight gain), cytogenetic (mitotic index-MI, micronucleus-MN and chromosomal abnormalities-CAs), biochemical (malondialdehyde-MDA, superoxide dismutase-SOD and catalase-CAT) and anatomical (root meristem cell damage) parameters. The genotoxicity mechanism of glyphosate was elucidated by spectral analysis. *A. cepa* bulbs were divided into six groups as one control and five applications. Tap water was applied to the bulbs in the control group for 72 h. Glyphosate (500 mg/L) and two different doses of Bmex (350 and 700 mg/L) were applied to the bulbs in the treatment group for 72 h. At the end of the period, the germinated bulbs were prepared for experimental analyses, measurements and observations by applying routine preparation procedures. As a result, glyphosate administration caused a significant (*p* < 0.05) decrease in all selected physiological parameter values, and significant (*p* < 0.05) increases in the number of cytogenetic parameters (except MI), the levels of biochemical parameters and the severity of anatomical damage. Glyphosate promoted CAs such as fragment, sticky chromosome, bridge and unequal distribution of chromatin in root tip meristem cells. By spectral analysis, it was determined that glyphosate interacts directly with DNA and causes genotoxicity. It also caused anatomical damages such as epidermis cell damage, cortex cell damage, flattened cell nucleus, binuclear cell and irregular vascular tissue in root tip meristem cells. Co-administration of glyphosate with Bmex at two different doses (350 and 700 mg/L) reduced the toxicity of glyphosate and led to significant (*p* < 0.05) improvements in the values of all parameters examined. It was determined that this improvement was even more pronounced at 700 mg/L dose of Bmex. As a result, it was determined that glyphosate herbicide caused multi-dimensional toxicity in *A. cepa* test material, and Bmex reduced the effects of this toxicity due to its antioxidant properties. Therefore, glyphosate dose ranges need to be reconsidered, especially considering non-target organisms in agricultural applications. In addition, antioxidant products such as Bmex should be included in the daily diet in order to reduce the toxic effects of environmental agents such as pesticides.

## Introduction

Synthesized chemicals or biological agents used to destroy any pest that causes damage to agricultural products are called pesticides. Pesticides are used to protect crops from insects, weeds and parasitic diseases during crop growth in agricultural areas. It is also applied to protect the harvested product against rodents, insects and various biological pollutants during storage. On the other hand, it is also used to clean unwanted aquatic plants, weeds, trees and bushes in roadsides, lakes and ponds. For this reason, pesticides are one of the synthetic chemicals that people encounter every day. Pesticides are chemicals that are deliberately released into nature. Pesticides from human activities can enter into water bodies via runoff and seepage. They can also be carried into the atmosphere by natural processes such as evaporation and wind. This leads to pollution of water, soil, flora and fauna^1^. Pesticides are classified according to various criteria. The most widely used criterion in the classification of pesticides is the classification made according to the target organism. According to this classification, pesticides are classified as acaricides, algicides, bactericides, fungicides, insecticides and herbicides^2^.

Herbicides are chemicals used to destroy weeds that damage agricultural crops. It is also used to destroy invasive weeds in wetlands. Rapid population growth and the need to produce food products in proportion to it have caused agricultural production activities to be dependent on herbicides. Herbicides account for more than 80% of the total pesticide consumption used to protect crops in agricultural areas. The vast majority of herbicides don't just target weeds^3^. At the same time, it can also affect non-target humans, plants and animals during the application. Herbicides degrade very slowly in nature due to their chemical structure. This situation not only causes herbicides to pollute the soil, water, environment and air but also brings serious health problems. The use of some herbicides is prohibited today, especially because they cause fatal health problems such as cancer^4^.

Glyphosate [(HO)_2_P(O)CH_2_NHCH_2_CO_2_H] is the most widely used herbicide worldwide today. Glyphosate is an organophosphorus herbicide. It has high solubility in water. It is a non-selective herbicide widely used against annual and perennial weeds in agricultural areas, home gardens and urban areas. Especially since it is applied intensively to agricultural lands, its residues can be found in water, soil, plants, food products and human urine. Glyphosate is classified in category 2a as a possible human carcinogen according to the International Agency for Research on Cancer (IARC)^5,6^. The herbicidal effect of glyphosate is based on the inhibition of the enzyme 5-enolypyruvilshikimate-3-phosphate synthase (EPSP), which is produced in plants, fungi and some microorganisms. EPSP synthase is a monomeric enzyme with a molecular mass of approximately 46,000. It participates in the biosynthesis of the aromatic amino acids phenylalanine, tyrosine and tryptophan via the shikimate pathway. When glyphosate enters the cell, it selects the EPSP enzyme as its biological target. Glyphosate binds to the EPSP enzyme, leading to its inhibition and blocking the shikimate pathway. As a result, the organism cannot synthesize the aromatic amino acids it needs to survive and the organism dies. There is also information in the literature that glyphosate inhibits the acetyl-cholinesterase enzyme and induces oxidative stress^6,7^.

In recent years, there has been an increase in the use of herbal products in studies carried out to reduce pesticide-induced toxicity. One of these products is bitter melon. Bitter melon is a shrub-shaped plant that grows in Bangladesh, India, China, Korea and Asian countries, as well as tropical regions of the Amazon, East Africa and the Caribbean. It belongs to the Cucurbitaceae family and is known by the scientific name *Momordica charantia* L. Bitter melon fruits are used as culinary vegetables in some countries. However, in many countries, it is used as a medicinal plant in the treatment of various diseases. It is widely used especially for the treatment of diabetes^8,9^. It is also preferred in the treatment of diseases such as eczema, rheumatism, obesity, leprosy, gout, psoriasis, kidney stones, jaundice, malaria, hemorrhoids, scabies and hypertension. Scientific studies carried out in recent years have shown that this plant has antibacterial, antiviral and anticarcinogenic activities. Many biologically active molecules such as glycosides, saponins, alkaloids, fixed oils, proteins, steroids, triterpenes and phenolic compounds in the content of *M. charantia* play a role in these activities. The main phenolic compounds found in *M. charantia* are gallic acid, tannic acid, catechin, caffeic acid, p-coumaric, gentisic acid, chlorogenic acid and epicatechin. Charantin, kuguasins, momordisin, and caravilagenines are the main triterpenoids found in *M. charantia*^10^.

The increasing use of glyphosate in agricultural areas and its ability to reach many non-target organisms through the food chain has raised increasing concerns about the toxicity of this herbicide. For this reason, the number of studies on the toxic effects of glyphosate has increased considerably in recent years. In this study, the toxic effects of glyphosate herbicide were discussed in all aspects with the help of physiological, cytogenetic, biochemical and anatomical parameters, and the mechanism of genotoxicity was also clarified by spectral shift analysis. Natural products have a very important role in reducing the effects of toxic agents. In this study, the toxicity-reducing effect of bitter melon extract (Bmex) was investigated depending on the dose. The dose-related regression of toxicity in all tested parameters was examined and the recovery effect of Bmex was calculated.

## Materials and methods

### Experimental design

In this study, the toxicity of glyphosate and the recovery effects of Bmex were investigated with the *Allium* test. This test is a reliable, simple, easy to perform test for toxicity analysis. It also shows a high correlation with eukaryotic test systems, bone marrow toxicity tests and results performed in rodents^11–13^. *A. cepa* bulbs (2n = 16) were used as test material, glyphosate (Merck, CAS No: 1071-83-6) as a toxic agent and bitter melon extract (Bmex) (Yesilex, 700 mg × 60 capsules) as a protective biological product. The content of Bmex consists entirely of natural extract obtained from the fruit of the plant. Commercially available Bmex capsules contain only plant extract and no additives.

All processes related to bulbs were carried out in accordance with international standards for the use of plants in experiments^14,15^. *Allium* bulbs were kept in 70% alcohol for 30 s and 1% sodium hypochlorite for 1 min, then washed twice with sterile distilled water to ensure surface sterilization. *A. cepa* bulbs are divided into 6 groups as Group I: control, Group II: 350 mg/L Bmex, Group III: 700 mg/L Bmex, Group IV: 500 mg/L glyphosate, Group V: 500 mg/L glyphosate + 350 mg/L Bmex, Group VI:500 mg/L glyphosate + 700 mg/L Bmex. Bulbs were germinated in sterile glass beakers for 72 h at room temperature. Tap water was applied to the bulbs in the control group and 500 mg/L glyphosate, 350 and 700 mg/L doses of Bmex were applied to the bulbs in the treatment groups. During the 72 h of germination, the beakers were checked daily and the decreasing solution was added. At the end of the period, the bulbs were washed in distilled water and made ready for physiological, cytogenetic, biochemical and anatomical examinations by applying the accepted routine preparation techniques^16^.

In this study, a dose of 500 mg/L glyphosate, which was used in a previous study by Çavuşoğlu et al.^17^ and known to be toxic, was preferred. Bmex doses have been determined by nutritionists considering the dose range recommended by nutritionists in the daily diet, as well as the dose range where it exhibits a protective feature against various toxicity^18,19^.

**Figure 1:**
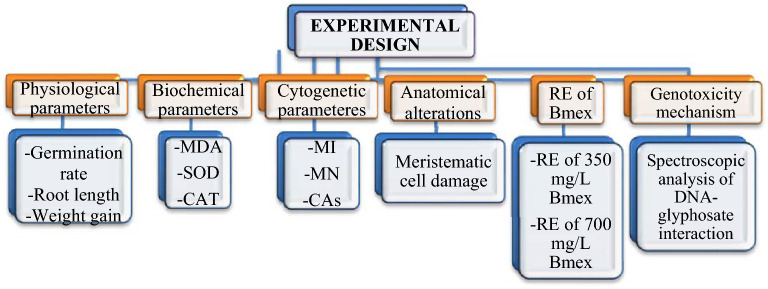
Experimental design of the study. MDA: malondialdehyde, SOD: superoxide dismutase, CAT: catalase, MI: mitotic index, MN: micronucleus, CAs: chromosomal abnormalities, RE: Recovery effect.

Experimental research on plant samples, including the supply of plant material, complies with institutional, national and international guidelines and legislation. To determine the glyphosate toxicity and the protective effect of Bmex, different parameters were examined in root tip meristematic cells, and the analyzed parameters are given in Fig. 1.

### Physiological parameter measurements

The inhibitory effects of glyphosate against selected physiological parameters and the protective role of Bmex against these effects were determined according to the following criteria.

- Root length was determined by measuring the length of the radicle, which is the embryonic root zone and forming the roots in the mature plant with a ruler (*mm*).
- Weight gain was determined by weighing the bulb weights with a precision balance before and after the experimental application,
- Germination percentage was determined with the help of Eq. (1)^20^.
  1 $$ {\text{Germination }}\left( \% \right) = \left[ {number \, of \, germinated \, seeds/total \, number \, of \, seeds} \right] \times {1}00 $$

50 bulbs in each group were used for germination percentage and weight gain. For root length measurements 10 bulbs were used in each group. A total of five measurements were taken from each bulb and a total of 50 measurements were obtained for one group for root length.

### Cytogenetic observations

To detect CAs and the presence of MN, root tips were cut about 1 cm in length, fixed in Clarke fixator consisting of ethyl alcohol (3 volumes) and glacial acetic acid (1 volume) for 2 h and washed in ethyl alcohol (96%) for 15 min. Root tips were hydrolyzed in 1 N HCl at 60 °C for 17 min. After hydrolysis, root tips were soaked in glacial acetic acid (45%) for 30 min. Finally, the root tips were stained with acetocarmine for 24 h, crushed on a slide, covered with a coverslip and examined under the Irmeco IM-450 TI model research microscope^21^. CAs and MN counts were made by two different observers and photographed at × 500 magnification. 1000 cells in each group were analyzed for MN and CAs.

MN determination was made according to the criteria determined by Fenech et al.^22^. These:

- The diameter of the MN should be approximately 1/3 of the cell nucleus.
- MN should be round or oval.
- In case of contact of MN and cell nuclear membranes, the boundary between them should be clearly distinguishable.

MI, which shows the ratio of cells undergoing mitosis to all cells, was calculated with the help of Eq. (2). 10,000 cells in each group were analyzed for MI.

2 $$ {\text{MI }}\left( \% \right) = \left[ {number \, of \, cells \, undergoing \, mitosis/total \, number \, of \, cells} \right] \times {1}00 $$

### Confirmation of the DNA-glyphosate interactions by spectral measurements

To elucidate the genotoxicity mechanism of glyphosate, the UV spectrum of the DNA-Glyphosate complex was investigated. For this purpose, DNA was first obtained from *A. cepa* root tip cells. DNA isolation from *Allium* root samples was carried out according to the method suggested by Sharma et al.^23^. 2 g of fresh root tips were crushed in liquid nitrogen. Crushed root tips were transferred to a polypropylene tube. 5 M NaCl and 2% sarcosyl (*5 mL*) were added to tube and shaken slowly. After centrifuging at 6800 g at room temperature for 15 min, the supernatant was transferred to a new polypropylene tube and an equal volume of chloroform-isoamylalcohol (24:1) was added and then mixed by inversion for 1 min. The solution was centrifuged at 3.200 g at room temperature for 15 min and the supernatant was carefully transferred to a new tube. 2 volumes of freshly prepared extraction buffer (100 mM Tris–Cl-pH 8, 20 mM EDTA, 1.4 M NaCl, 2%CTAB) were added to the supernatant and mixed by inversion. After incubation for 35 min at 100 rpm in a shaking water bath at 60 °C, an equal volume of chloroform-isoamylalcohol (24:1) was added and mixed gently. After centrifugation, the upper aqueous layer was carefully removed with a pipette and 3 M sodium acetate (pH 5.2) was added in 1/30 volume. The sample was centrifuged at 3200 g for 10 min at room temperature after mixing with isopropanol. The supernatant was discarded and the pellet was washed with 80% ethanol. The pellet was air-dried for 30 min and dissolved in 0.5 mL of TE buffer (10 mM Tris–Cl-pH 8 and 1 mM EDTA). 5 µL of RNase A (10 mg/mL) was added and incubated at 37 °C for 30 min. It was extracted with an equal volume of chloroform-isoamylalcohol (24:1). The aqueous layer was transferred to a new 0.5 mL microfuge tube and 2 volumes of cold ethanol were added. It was centrifuged at 3200 g for 10 min at room temperature. The pellet was washed with 80% ethanol. The pellet was dried and dissolved in 300 µL of distilled water and stored a 4 °C.

DNA-glyphosate interaction was evaluated by investigating the change in absorbance of mixtures containing DNA and different concentrations of glyphosate (1:1, 1:2, 1:4). The UV absorption spectrum of DNA-glyphosate complex in the range of 220–300 nm was obtained. UV absorption spectra were recorded on the Mapada UV-6100PCS double beam spectrophotometers.

### Biochemical measurements

#### Oxidative stress measurement (MDA)

MDA measurement was carried out according to the method suggested by Unyayar et al.^24^. 0.5 g of freshly harvested root tips were homogenized in 1 mL of trichloroacetic acid (5%) solution. The homogenate was transferred to a sterile test tube and centrifuged at 12,000 g for 10 min. The supernatant and thiobarbituric acid (0.5%) were transferred in equal volumes to a new sterile test tube and incubated in trichloroacetic acid solution (20%) at 96 °C for 30 min. At the end of the period, the test tube was placed in an ice bath and centrifuged at 10,000 g for 5 min. The absorbance of the supernatant was measured at 532 nm and the MDA level was expressed as μM/g FW. MDA levels were measured in triplicate and repeated three times.

#### Antioxidant enzyme activity measurements

Enzyme extraction was carried out at + 4 °C. 0.5 g of freshly harvested root tips were washed with distilled water, homogenized in 5 mL of monosodium phosphate buffer (50 mM, pH 7.8), centrifuged at 10,500 g for 20 min, and the supernatant stored at + 4 °C until analysis^25^. SOD and CAT measurements were performed in triplicate and repeated three times.

#### SOD measurement

SOD measurement was carried out according to the method proposed by Beauchamp and Fridovich^26^. A total volume of 3 mL of reaction solution (1.5 mL 0.05 M monosodium phosphate buffer, 0.3 mL 130 mM methionine, 0.3 mL 750 μM nitroblue tetrazolium chloride, 0.3 mL 0.1 mM EDTA-Na_2_, 0.3 mL 20 μM riboflavin, 0.28 mL deionized water, 0.01 mL enzyme extract, 0.01 mL 4% insoluble polyvinylpyrrolidone) was prepared. The reaction was started by placing the tubes under two 15 W fluorescent lamps for 10 min and was terminated by keeping the tubes in the dark for 10 min. Absorbance was measured at 560 nm and SOD activity was expressed as U/mg FW^25^.

#### CAT measurement

CAT measurement was carried out according to the method proposed by Beers and Sizer^27^. CAT activity was measured in a UV–VIS spectrophotometer at room temperature by preparing a total volume of 2.8 mL reaction solution (0.3 mL 0.1 M H_2_O_2_, 1.0 mL dH_2_O, 1.5 mL 200 mM monosodium phosphate buffer). The reaction was started with the addition of 0.2 mL of enzyme extract. CAT activity was measured by monitoring the decrease in absorbance at 240 nm as a result of H_2_O_2_ consumption and expressed as OD240 nm min./g^25^.

### Observation of meristematic cell damages

Root tips cut in 1 cm length were washed with distilled water, placed between soft foam material and cross-sections were taken with the help of a sterile razor blade. Sections were placed on a slide, stained with methylene blue (5%) for 2 min, covered with a coverslip and examined under the Irmeco IM-450 TI model research microscope and photographed at × 200 magnification^28^.

### Recovery effects of Bmex

In the determination of the recovery effects (RE) of Bmex, the data of Group V and VI, the data of the 500 mg/L glyphosate treated group and the data of the control group were taken as the basis. RE of Bmex was calculated using the following Eq. (3).

3 $$ {\text{Recovery effect }}\% = \left[ {\left( {D_{1} - D_{2} } \right) \, /\left( {D_{3} - D_{2} } \right)} \right] \times {1}00 $$

D_1_: data of 500 mg/L glyphosate + 350 mg/L or 700 mg/L Bmex treated group, D_2_: data of 500 mg/L glyphosate treated group, D_3_: data of control group.

### Statistical analysis

Statistical evaluation was made with the help of SPSS Statistics 22 (IBM SPSS, Turkey) package program. Data are shown as mean ± standard deviation (SD). Statistical significance between the means was determined using one-way analysis of variance (One-way ANOVA) and Duncan tests. It was considered statistically significant when the *p* value obtained was < 0.05.

## Results and discussion

### Physiological effects

The effects of glyphosate and Bmex application on selected physiological parameters are shown in Table 1. The highest germination percentage, root length and weight gain were determined in the control group and in group II and group III, where two different doses of Bmex (350 and 700 mg/L) were applied. There was no statistically significant difference (*p* > 0.05) between the physiological parameter values measured in these groups. Glyphosate administration at a dose of 500 mg/L resulted in statistically significant (*p* < 0.05) decreases in all selected physiological parameters. Compared to the control group, this reduction in group IV was 53% for germination, about 2.65 times for root length, and about 3.46 for weight gain. Co-administration of glyphosate and Bmex again caused statistically significant (*p* < 0.05) increases in the values of the selected physiological parameters. It has been determined that these increases are directly proportional to the dose of Bmex. Compared to group IV, these increases were 24% for germination, 1.74 times for root length and 2.52 times for weight gain in group VI, where 700 mg/L of Bmex was applied.

**Table 1:** Protective role of Bmex against the physiological changes induced by glyphosate.

Groups	Germination (%)	Root length (cm)	Initial weight (g)	Final weight (g)	Weight gain (g)
Group I	98^a^	6.10 ± 0.85^a^	6.48 ± 0.94	11.84 ± 1.44	+ 5.36^a^
Group II	100^a^	5.80 ± 0.81^a^	6.60 ± 0.96	11.88 ± 1.42	+ 5.28^a^
Group III	99^a^	6.30 ± 0.87^a^	6.42 ± 0.92	11.89 ± 1.45	+ 5.47^a^
Group IV	45^d^	2.30 ± 0.42^d^	6.50 ± 0.95	8.05 ± 1.06	+ 1.55^d^
Group V	54^c^	3.10 ± 0.48^c^	6.65 ± 0.98	9.39 ± 1.12	+ 2.74^c^
Group VI	69^b^	4.00 ± 0.56^b^	6.45 ± 0.94	10.35 ± 1.17	+ 3.90^b^

Group I: Control, Group II: 350 mg/L Bmex, Group III: 700 mg/L Bmex, Group IV: 500 mg/L glyphosate, Group V: 500 mg/L glyphosate + 350 mg/L Bmex, Group VI: 500 mg/L glyphosate + 700 mg/L Bmex. Values are shown as mean ± SD. 50 bulbs in each group were used for germination percentage and weight gain. For root length measurements 10 bulbs were used in each group. The averages shown with different letters^(a–d)^ in the same column are significant at *p* < 0.05.

There are some studies in the literature investigating the physiological toxicity induced by glyphosate in different plant species. For example, Çavuşoğlu et al.^17^ reported that administration of glyphosate at doses of 100, 250 and 500 mg/L caused a dose-dependent decrease in germination, root length and weight gain in *A. cepa*. Kamdem et al.^29^ observed that plant height, root length, leaf number, stem diameter, leaf surface, chlorophyll amount and dry matter yield decreased in *Zea mays* L. (*corn*) and *Phaseolus vulgaris* L. (*bean*) plants grown in soil treated with six different doses (0.1, 0.2, 0.4, 0.6, 0.8 and 1.0 g/kg) of glyphosate, depending on the application dose and duration (21, 28, 35 and 42 days). Grzesiuk et al.^30^ found a decrease in root length and root, cotyledon and hypocotyl weights of radish (*Raphanus sativus* L.) seedlings exposed to 0.1, 0.5 and 2.0 mM concentrations of glyphosate for 4, 7 and 14 days. In this study, the decrease in physiological parameters and germination observed as a result of glyphosate application is thought to be due to the inhibition of water and mineral substance intake. Glyphosate strongly inhibits germination, root growth and other processes in plants by reducing/inhibiting the absorption and displacement of macro and micro elements such as Ca, Mg, Mn and Fe^31^. In addition, by blocking the production of tryptophan, glyphosate inhibits the synthesis of indole acetic acid, a growth promoter that plays an important role in root growth of plants^32^. Another reason for reducing root elongation and germination may be that glyphosate inhibits mitotic cell division in stem cells. It has been reported in the literature that root elongation in plants is directly related to the frequency of mitosis observed in root meristem cells^33^. Glyphosate may cause regression in physiological processes by inhibiting cell cycle and mitotic division through malformation of the mitotic spindle and incorrect attachment of chromosomes to spindles.^34^.

### Cytogenetic effects

**Figure 2:**
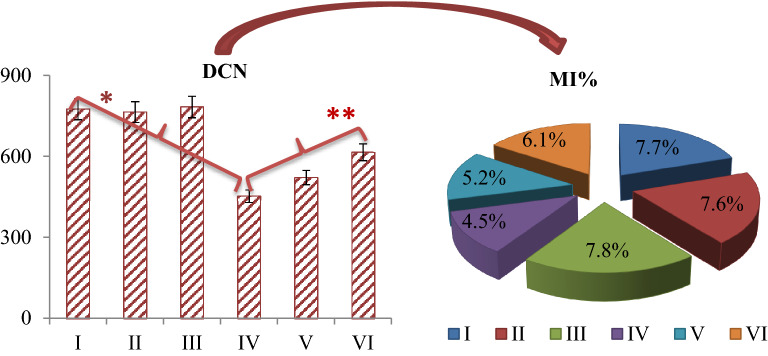
Bmex and glyphosate effects on dividing cell number (DCN) and %MI in *A. cepa* root tip cells. Group I: control, Group II: 350 mg/L Bmex, Group III: 700 mg/L Bmex, Group IV: 500 mg/L glyphosate, Group V: 500 mg/L glyphosate + 350 mg/L Bmex, Group VI: 500 mg/L glyphosate + 700 mg/L Bmex. *indicates the statistical difference between Groups I and IV, **indicates a statistical difference between Groups IV and VI (*p* < 0.05). 10,000 cells in each group were analyzed for MI.

**Figure 3:**
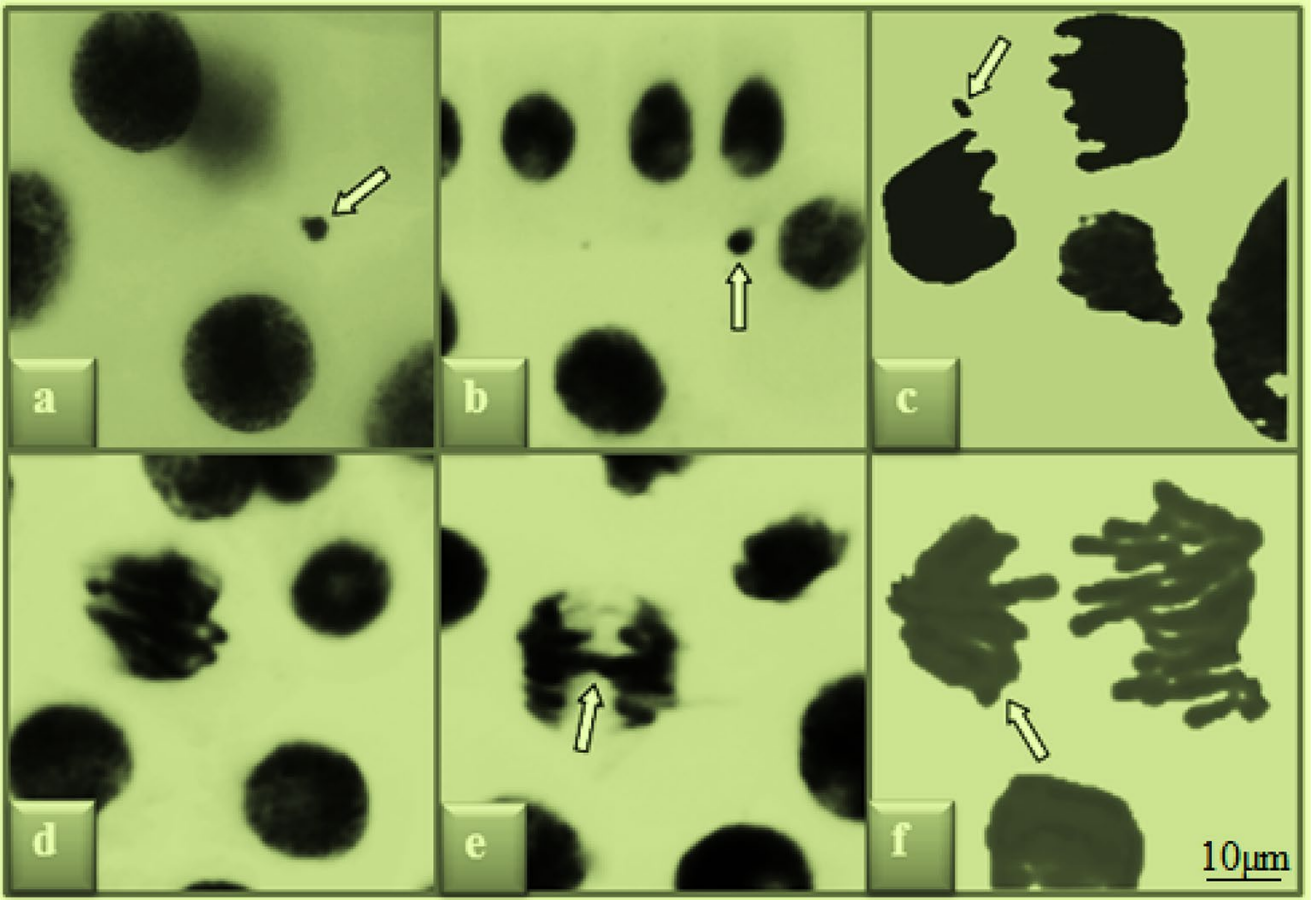
Chromosomal abnormalities induced by glyphosate. MN in interphase (**a**,**b**), fragment in telophase (**c**), sticky chromosome in prophase (**d**), bridge in anaphase (**e**), unequal distribution of chromatin in anaphase (**f**).

The effects of glyphosate and Bmex application on selected cytogenetic parameters are shown in Figs. 2 and 3 and Table 2. The highest MI value and the lowest MN and CAs numbers were determined in the control group, group II and group III, where two different doses of Bmex (350 and 700 mg/L) were administered. No damage was observed in these groups, except for a few MN and sticky chromosome, which were not statistically significant (*p* > 0.05). In addition, there was no statistically significant difference (*p* > 0.05) between the MI values determined in these groups. Glyphosate administration at a dose of 500 mg/L caused a decrease in MI and an increase in the number of MN and CAs in group IV. It was determined that this increase and the decrease were statistically significant (*p* < 0.05). Glyphosate administration promoted CAs such as fragment, sticky chromosome, bridge, and unequal distribution of chromatin in root tip meristem cells. The combination of glyphosate and Bmex caused a statistically significant (*p* < 0.05) increase in MI values and a statistically significant (*p* < 0.05) decrease in MN and CAs numbers again. It has been determined that these increases and decreases are directly related to the dose of Bmex. Compared to group IV, MI increased 1.63% in group VI, MN decreased approximately 1.49 times and fragment (most observed chromosomal damage) decreased approximately 1.45 times.

**Table 2:** Protective role of Bmex against glyphosate-induced genotoxicity.

Abnormalities	Group I	Group II	Group III	Group IV	Group V	Group VI
MN	0.16 ± 0.40^d^	0.12 ± 0.30^d^	0.00 ± 0.00^d^	61.7 ± 4.58^a^	50.8 ± 3.96^b^	41.4 ± 3.75^c^
FRG	0.00 ± 0.00^d^	0.00 ± 0.00^d^	0.00 ± 0.00^d^	54.6 ± 3.98^a^	46.5 ± 3.84^b^	37.6 ± 3.62^c^
SC	0.24 ± 0.50^d^	0.20 ± 0.42^d^	0.17 ± 0.41^d^	45.3 ± 3.80^a^	35.7 ± 3.55^b^	26.8 ± 2.86^c^
B	0.00 ± 0.00^d^	0.00 ± 0.00^d^	0.00 ± 0.00^d^	36.8 ± 3.57^a^	28.4 ± 2.90^b^	19.7 ± 1.98^c^
UDC	0.00 ± 0.00^d^	0.00 ± 0.00^d^	0.00 ± 0.00^d^	24.3 ± 2.75^a^	16.6 ± 1.78^b^	9.70 ± 0.96^c^

Group I: Control, Group II: 350 mg/L Bmex, Group III: 700 mg/L Bmex, Group IV: 500 mg/L glyphosate, Group V: 500 mg/L glyphosate + 350 mg/L Bmex, Group VI: 500 mg/L glyphosate + 700 mg/L Bmex. Values are shown as mean ± SD (*n* = *10*). 1000 cells in each group were analyzed for MN and CAs. The averages shown with different letters^(a–d)^ in the same line are significant at *p* < 0.05.

*MN* micronucleus, *FRG* fragment, *SC* sticky chromosome, *B* bridge, *UDC* unequal distribution of chromatin.

There are similar studies in the literature investigating glyphosate genotoxicity in non-target plants. For example, Çavuşoğlu et al.^17^ reported that three different doses (100, 250 and 500 mg/L) of glyphosate caused a dose-dependent decrease in MI, an increase in MN frequency, and CAs such as fragments, sticky chromosome, bridge, and unequal distribution of chromatin in *A. cepa* root meristem cells. Mercado and Caleño^35^ found a dose-related decrease in MI and a dose-related increase in the frequency of MN in *A. cepa* root tip cells exposed to glyphosate at 5, 10, 15, 25 and 30 mg/L doses. They also observed that glyphosate induced CAs such as bridge, disordered mitosis, break and nuclear elongation.

In this study, it is thought that the increases observed in the number of MN and CAs in *A. cepa* root tip meristem cells as a result of glyphosate application occur as a result of direct or indirect binding of glyphosate to DNA (by creating ROS). Because there are some studies in the literature that glyphosate directly interacts with DNA and causes nucleoplasmic bridge formation^36^ and DNA methylation^37^, and also affect genes in DNA by creating oxidative stress through ROS^38^. The decrease observed in the MI values of root cells suggests that glyphosate may be due to the inhibition of microtubule synthesis, which is responsible for chromosome migration during cell division. Because there are some studies in the literature that herbicides bind to plant tubulin protein, inhibit microtubule polymerization and reduce MI^39^.

### DNA-glyphosate interaction confirmed by the spectral shift

**Figure 4:**
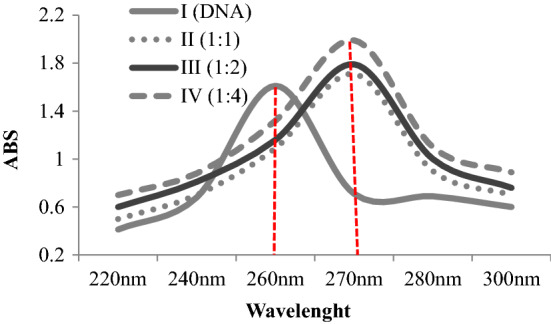
Bathochromic and hyperchromic shifts in the UV spectrum of DNA after interaction with glyphosate.

The interactions of glyphosate with different DNA sequences have been investigated by various methods in the literature. It has been reported in the literature that glyphosate directly interacts with DNA and causes different abnormalities^36^. CAs and MN formations induced by glyphosate in this study may also be associated with DNA-glyphosate interaction. To confirm this relationship, the spectral shift profile in UV absorption spectrum of *Allium*-DNA was investigated and the results are given in Fig. 4. The UV–VIS spectrum of *Allium-*DNA showed a characteristic maximum peak at ∼ 260 nm. The addition of glyphosate to DNA solution caused bathochromic and hyperchromic shifts in the UV spectrum. The bathochromic shift was from 260 nm to approximately 270 nm, and the hyperchromic shift was from 1.61 to 1.99. As the glyphosate ratio increased, the shift intensity also increased. Hyperchromicity is defined as an increase in the absorbance of a material, and the most well-known example is the hyperchromicity of DNA that occurs due to helical structure denaturation^40^. Spectral analyzes indicate the interaction of glyphosate with DNA. This interaction can cause DNA dissociation, disruption of its integrity, chain breaks, MN and CAs formations. The high-frequency of CAs and MN observed in this study may be caused by DNA-glyphosate interaction and the mechanism of genotoxicity can be explained by this interaction.

### Biochemical effects

**Figure 5:**
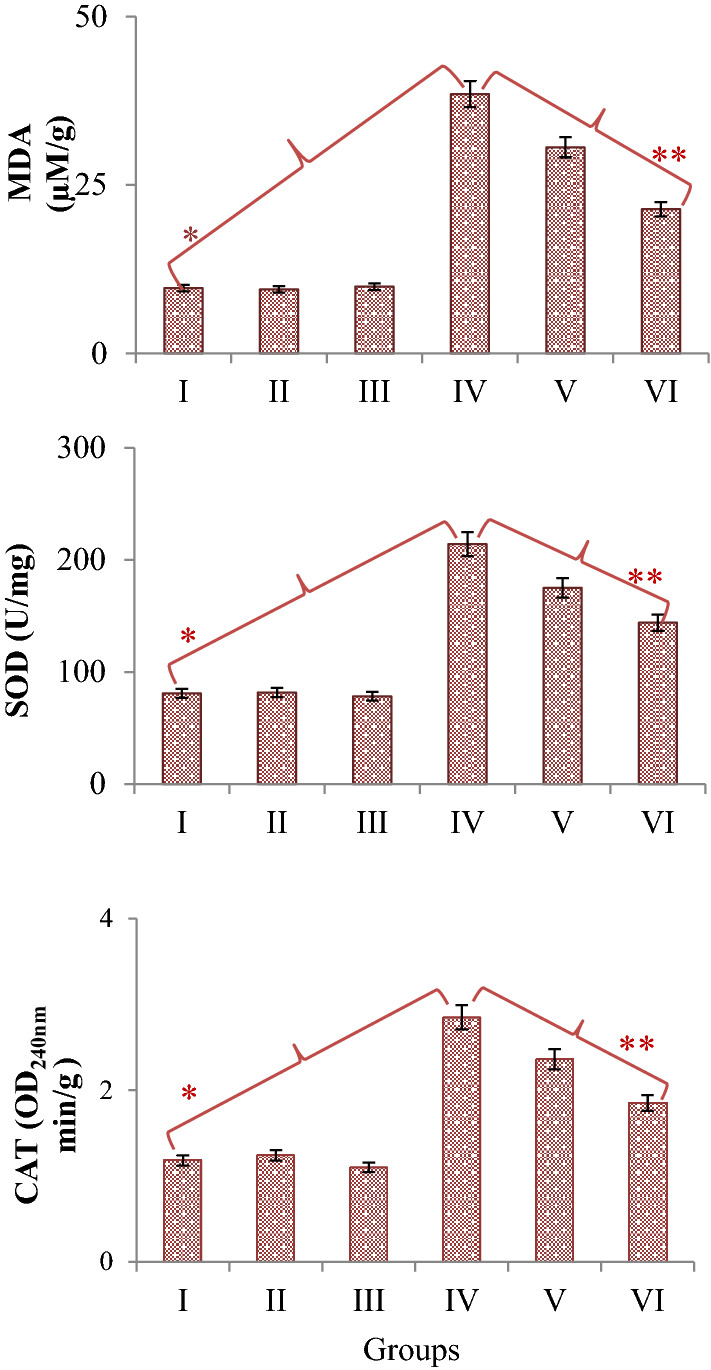
Protective role of Bmex against the biochemical changes induced by glyphosate. Group I: Control, Group II: 350 mg/L Bmex, Group III: 700 mg/L Bmex, Group IV: 500 mg/L glyphosate, Group V: 500 mg/L glyphosate + 350 mg/L Bmex, Group VI: 500 mg/L glyphosate + 700 mg/L Bmex. Values are shown as mean ± SD (*n* = 10). *indicates the statistical difference between Groups I and IV, **indicates a statistical difference between Groups IV and VI (*p* < 0.05).

The effects of glyphosate and Bmex application on selected biochemical parameters are shown in Fig. 5. The lowest MDA level, SOD and CAT enzyme activities were measured in the control group and group II and group III, which were administered two different doses (350 and 700 mg/L) of Bmex. There was no statistically significant difference (*p* > 0.05) between the measured biochemical parameter values in these groups. Glyphosate administration at a dose of 500 mg/L caused a statistically significant (*p* < 0.05) increase in all selected biochemical parameter values in Group IV. Compared to the control group, MDA level increased approximately 3.97 times, SOD enzyme activity approximately 2.65 times and CAT activity approximately 2.42 times in Group IV. The combined application of glyphosate and Bmex again caused a statistically significant (*p* < 0.05) decrease in the selected biochemical parameter values. It was determined that these decreases were more pronounced at 700 mg/L dose of Bmex. Compared to group IV, these decreases were 1.80 times for MDA level, 1.49 times for SOD enzyme activity and 1.54 times for CAT enzyme activity in group VI, where 700 mg/L dose of Bmex was administered.

There are a limited number of studies in the literature investigating lipid peroxidation and oxidative stress caused by glyphosate in plant cells. For example, Çavuşoğlu et al.^17^ reported that administration of glyphosate at 100, 250 and 500 mg/L doses caused a dose-dependent increase in MDA levels in *A. cepa* root cells and promoted lipid peroxidation. Akbulut et al.^41^ determined that peroxidase (POD), ascorbate peroxidase (APX), glutathione-S-transferase (GST), SOD and CAT enzyme activities increased in *Z. mays* seeds applied glyphosate in the 0.017–0.145 M concentration range on the 1st, 5th and 10th days. They also observed that these increases were directly proportional to the application dose and duration. Shopova et al.^42^ detected significant increases in the levels of MDA, which are markers of oxidative damage, and in SOD and CAT enzyme activities in the leaves of winter wheat (*Triticum aestivum* L.) seedlings sprayed with glyphosate.

MDA is the main metabolite formed as a result of the oxidation of membrane lipids, especially under the influence of free radicals, and is used as a marker of oxidative stress. It can bind (covalently or crosswise) to lipids, proteins, RNA and DNA in the cell^43^. In this study, it is thought that the increase observed in root MDA levels as a result of glyphosate application is due to the fact that glyphosate accelerates lipid destruction by damaging cell membranes. Because it has been reported in the literature that glyphosate causes oxidative stress in plant cells, disrupts membrane integrity, and ultimately promotes lipid peroxidation, causing an increase in the amount of MDA^41^.

SOD and CAT are the main antioxidant enzymes. They protect the cell against the harmful effects of free radicals. SOD also takes part in the first-line defense developed by the cell against free radicals. It is an enzyme that catalyzes the conversion of superoxide radicals to H_2_O_2_ and oxygen. The resulting H_2_O_2_ is converted into water and oxygen by another antioxidant enzyme, CAT. As a result, the detoxification process initiated by SOD in the cell is completed by CAT^44^. In this study, it is thought that the increases observed in SOD and CAT enzyme activities as a result of glyphosate application, glyphosate cause may excessive free radical production in the cells and the cells increase the SOD and CAT enzyme levels to destroy these free radicals formed. De Freitas-Silva et al.^45^ reported that glyphosate administration increased oxidative stress, oxidized proteins, induced antioxidant enzymes, and increased CAT activity in *Arabidopsis thaliana.*

### Anatomical effects

**Figure 6:**
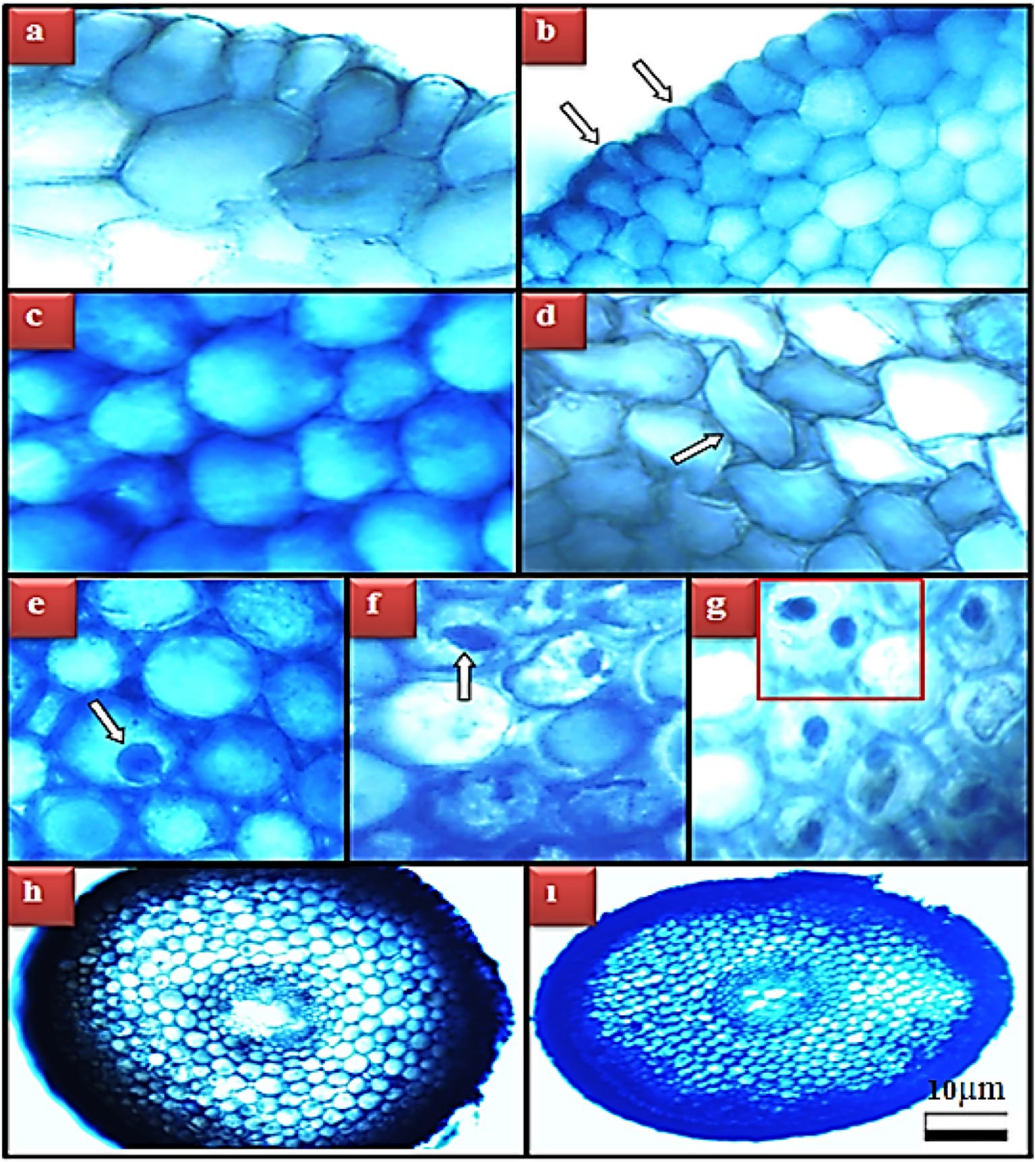
Meristematic cell damages induced by glyphosate. Normal appearance of epidermis cells (**a**), epidermis cell damage (**b**), normal appearance of cortex cells (**c**), cortex cell damage (**d**), normal appearance of cell nucleus-oval (**e**), flattened cell nucleus (**f**), binuclear cell (**g**), normal appearance of the vascular tissue (**h**), irregular vascular tissue (**i**).

The damages induced by glyphosate application in root tip meristem cells of *A. cepa* are shown in Fig. 6 and Table 3. In the examinations performed under the microscope, no cell damage was observed in the control group and in group II and group III, where two different doses of Bmex (350 and 700 mg/L) were applied. Five different types of damage were observed in the root tip meristem cells of Group IV, which received glyphosate at a dose of 500 mg/L, such as epidermis cell damage, cortex cell damage, flattened cell nucleus, binuclear cell, and irregular vascular tissue. Co-administration of glyphosate and Bmex resulted in dose-dependent improvements in the severity of the five types of damage observed. Glyphosate-induced binuclear cell and irregular vascular tissue damage were not observed, especially in Group VI, where 700 mg/L of Bmex was administered.

**Table 3:** Protective role of Bmex against glyphosate-induced meristematic cell damage.

Groups	ECD	CCD	FCN	BC	IVT
Group I	–	–	–	–	–
Group II	–	–	–	–	–
Group III	–	–	–	–	–
Group IV	+++	+++	+++	++	++
Group V	++	++	++	+	+
Group VI	+	+	+	–	–

Group I: Control, Group II: 350 mg/L Bmex, Group III: 700 mg/L Bmex, Group IV: 500 mg/L glyphosate, Group V: 500 mg/L glyphosate + 350 mg/L Bmex, Group VI: 500 mg/L glyphosate + 700 mg/L Bmex. ECD: epidermis cell damage, CCD: cortex cell damage, FCN: flattened cell nucleus, BC: binuclear cell, IVT: irregular vascular tissue. (*–*): no damage, (+): little damage, (++): moderate damage (+++): severe damage.

There is no other comprehensive study in the literature investigating the anatomical damage induced by glyphosate in root meristem cells, apart from the study performed by our study team. In this study carried out by Çavuşoğlu et al.^17^ it was reported that administration at doses 100, 250 and 500 mg/L of glyphosate caused anatomical damages such as epidermis cell damage, cortex cell damage, flattened cell nucleus, binuclear cell and irregular vascular tissue in *A. cepa* root meristem cells. Although there is not much about glyphosate in the literature, there are some studies investigating the anatomical damage caused by other pesticides in *A. cepa*. For example, Çavuşoğlu et al.^46^ determined that exposure to 20 mg/L dose of spirodiclofen caused damage to *A. cepa* root tip meristem cells such as epidermis cell damage, thickening of the cortex cell wall, flattened cell nuclei and irregular vascular tissue. Yirmibeş et al.^47^ observed that 100 mg/L dose of paraquat herbicide application induced epidermis cell damage, cortex cell damage, flattened cell nucleus and irregular vascular tissue damage in *A. cepa* root tip meristem cells.

In this study, microscopic damages observed in root tip meristem cells of *A. cepa* as a result of glyphosate application suggest that they are caused by mechanical stress as a result of physical defense mechanisms developed by the organism to reduce the uptake of glyphosate into the cell. Because, in the examinations performed under the microscope, a significant increase was detected in the number and order of cells in the epidermis and cortex cells of the groups treated with glyphosate. This defense developed by the stem cells to prevent glyphosate from entering the cell increases the contact of the cells with each other and this causes a mechanical pressure. This pressure may cause deformities in the epidermis and cortex cells and the nucleus of these cells. The information in the literature that plants develop many chemical (such as phytoalexins-diterpenoids and phytoanticips-maysin)^48^ and physical (increases in the number and order of epidermis and cortex cells, thickening of the cell wall)^46^ defense mechanisms to protect them from pesticide toxicity supports this idea.

### Recovery effects of Bmex

**Figure 7:**
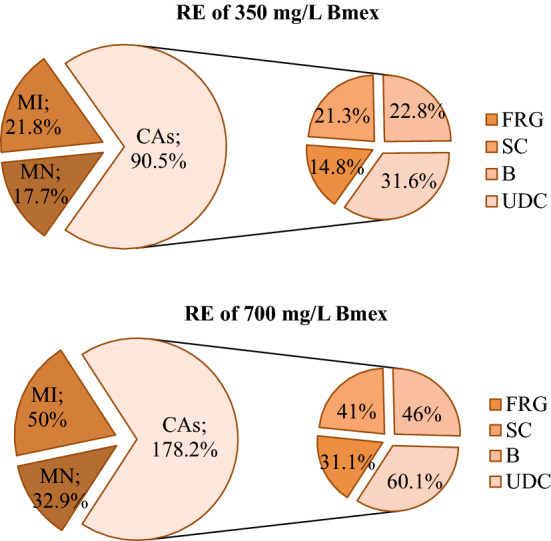
RE of 350 mg/L and 700 mg/L doses of Bmex against MI and CAs.

Bmex exhibited a toxicity-reducing effect in all tested parameters. In particular, it protected against the cytotoxic effects by reducing the MI rate, and against genotoxic effects by reducing the frequency of MN and CAs. 350 and 700 mg/L Bmex applications increased MI rates, providing 21.8% and 50% protection in MI against glyphosate cytotoxicity, respectively. There were significant decreases in MN and CAs frequencies in the groups treated with Bmex + glyphosate, and the most significant reduction was observed in Group VI, which was administered 700 mg/L Bmex. 350 mg/L Bmex reduced the CAs and MN frequencies induced by glyphosate in the range of 14.8–31.6%, while 700 mg/L Bmex reduced the frequency between 31.1 and 60.1% (Fig. 7).

In scientific studies carried out in recent years, plant extracts such as *Ginkgo biloba* L., grape seed, green coffee, green tea, sage, nettle are used to reduce or prevent toxicity caused by pesticides. In this study, Bmex was used to reduce the toxicity induced by glyphosate. Glyphosate administration caused changes in all selected physiological, cytogenetic, biochemical and anatomical parameters and induced toxicity. On the other hand, administration of 350 and 700 mg/L doses of Bmex reduced the toxicity induced by glyphosate and caused significant improvements in all parameters examined. It was determined that this improvement was more pronounced at 700 mg/L dose of Bmex. It is thought that many biologically active substances with antioxidant properties in the composition of Bmex play a role in this improvement. Because there are many minerals such as Fe, Mg, K and Mn, vitamin C and B complex vitamins such as niacin (*B*_3_), pantothenic acid (*B*_5_), pyridoxine (*B*_6_), fixed oils such as palmitic acid, palmitoleic acid, stearic acid, oleic acid, linoleic acid and linolenic acid, and glycosides, saponins, alkaloids, triterpenes, proteins, steroids in the structure of Bmex. All these active ingredients give antioxidant, antimicrobial, antiviral and anticarcinogenic properties to Bmex^49^. There are some studies in the literature investigating the protective role of Bmex in reducing the toxicity induced by different chemical agents, especially in animal organisms. For example; Abarshi et al.^50^ reported the protective role of Bmex on Pb-induced male reproductive toxicity in rats. Deng et al.^51^ determined the protective role of Bmex against liver damage in restraint-stressed mice. Kanpalta et al.^52^ observed that Bmex has a protective role against reproductive toxicity induced by methotrexate in male rats.

## Conclusion

This study is one of the most comprehensive studies dealing with the toxic effects of glyphosate with the help of multiple parameters. In addition, the protective role of Bmex in reducing glyphosate toxicity is also revealed in the study. As a result of this study, it was determined that glyphosate, which is the most preferred herbicide in the world, especially in the fight against invasive weeds in agricultural areas, causes a versatile toxicity in terms of physiological, cytogenetic, biochemical and anatomical. The application of Bmex decreased the toxicity of glyphosate depending on the dose and caused significant improvements in all the parameters examined. This study is the first to show that Bmex can be used to reduce glyphosate toxicity. Glyphosate is an herbicide that is widely used in agricultural applications and has an accumulation feature. Glyphosate has a binding affinity for soil particles and therefore accumulates mostly in the upper soil layers. After weeds die and rot, glyphosate can migrate between plants and be released into the rhizosphere. Cumulative glyphosate can also reach a wide range of non-target organisms by grazing animals or feeding humans. Considering its cumulative nature, the lowest doses as possible should be preferred, especially in the application of glyphosate in agricultural areas. Studies investigating the most effective dose for target organisms and non-toxic doses for non-target organisms are very valuable in determining these doses. On the other hand, the protective feature of antioxidant products such as Bmex should not be ignored in order to prevent or reduce the toxicity caused by pesticides.

## Data Availability

The datasets used and/or analyzed during the current study are available from the corresponding author on reasonable request.
